# Gallium Oxide Memristors: A Review of Resistive Switching Devices and Emerging Applications

**DOI:** 10.3390/nano15171365

**Published:** 2025-09-04

**Authors:** Alfred Moore, Yaonan Hou, Lijie Li

**Affiliations:** 1 Centre for Integrative Semiconductor Materials (CISM), Bay Campus, Swansea University, Swansea SA1 8EN, UK; 975288@swansea.ac.uk (A.M.); yaonan.hou@swansea.ac.uk (Y.H.); 2 Department of Electronic and Electrical Engineering, Bay Campus, Swansea University, Swansea SA1 8EN, UK

**Keywords:** capacitive switching, resistive switching, ON/OFF ratios, oxygen vacancy

## Abstract

Gallium oxide (Ga_2_O_3_)-based memristors are gaining traction as promising candidates for next-generation electronic devices toward in-memory computing, leveraging the unique properties of Ga_2_O_3_, such as its wide bandgap, high thermodynamic stability, and chemical stability. This review explores the evolution of memristor theory for Ga_2_O_3_-based materials, emphasising capacitive memristors and their ability to integrate resistive and capacitive switching mechanisms for multifunctional performance. We discussed the state-of-the-art fabrication methods, material engineering strategies, and the current challenges of Ga_2_O_3_-based memristors. The review also highlights the applications of these memristors in memory technologies, neuromorphic computing, and sensors, showcasing their potential to revolutionise emerging electronics. Special focus has been placed on the use of Ga_2_O_3_ in capacitive memristors, where their properties enable improved switching speed, endurance, and stability. In this paper we provide a comprehensive overview of the advancements in Ga_2_O_3_-based memristors and outline pathways for future research in this rapidly evolving field.

## 1. Introduction

**Figure 1:**
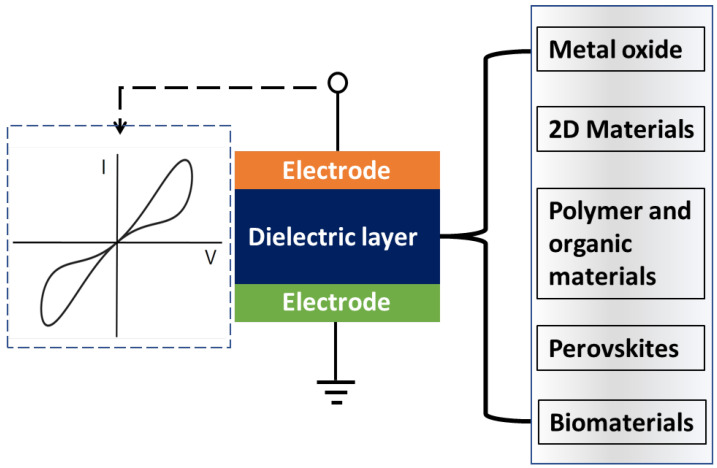
Schematic diagram showing various dielectric materials used in memristor devices.

The evolution of electronic devices has driven the demand for innovative materials and architectures capable of achieving superior performance, scalability, and energy efficiency. Among these, memristors have emerged as a promising technology, with their unique ability to combine memory and computation in a single nanoscale device [1]. Memristors, or memory resistors, were firstly theorised in 1971 by Leon Chua as the fourth fundamental circuit element, completing the relationship between electrical variables in circuit theory. Since their conceptualisation, memristors have evolved into a practical technology with applications in non-volatile memory, neuromorphic computing, and adaptive circuits. Resistive-switching memories (often termed RRAM or memristors) have emerged as leading candidates for non-volatile storage and neuromorphic computing because they combine two-terminal simplicity, analogue programmability, and CMOS compatibility. A broad spectrum of material platforms has been explored. Binary oxides such as TiO_2_, HfO_2_, and NiO [2] remain the most mature thanks to straightforward integration and well-studied vacancy-driven (VCM) and filamentary mechanisms [3]. Perovskite oxides provide rich defect chemistry and tunable electronic structures that enable low-voltage switching and analogue weight updates for synaptic operation [4]. Chalcogenide systems offer multi-level states but typically rely on thermally assisted structural/electronic transitions that can raise energy cost and variability [5]. Organic and polymer memories bring mechanical flexibility and solution processing, albeit with endurance and retention trade-offs relative to inorganic counterparts [6,7]. Nitrides (e.g., SiN_*x*_, AlN, GaN) are attractive for their strong bonds, thermal robustness, and compatibility with back-end processes [8]. Finally, 2D materials (graphene derivatives, TMDs, h-BN, etc.) enable ultimate thickness scaling, interfacial engineering, and low-energy switching pathways, though wafer-scale uniformity remains a challenge [9]. Recent surveys emphasise that no single material class dominates across all figures of merit - endurance, speed, retention, variability, and analogue linearity - so comparative benchmarking is essential when proposing a focus material [9]. Figure 1 schematically shows a memristor made of various dielectric materials. Against this backdrop, gallium oxide (Ga_2_O_3_) stands out as an ultra-wide-bandgap oxide with high breakdown field, thermal/chemical stability, and notable defect tolerance, which together suggest promise for high-temperature operation, low leakage, and robust cycling in memristors. Ga_2_O_3_ devices can leverage oxygen-vacancy switching with inert electrodes or (ElectroChemical Metallisation) ECM-type filament formation with active metals, while also enabling transparent or lateral device geometries via ITO and interdigitated contacts. In this review we synthesize the state of Ga_2_O_3_-based memristors. We discuss mechanisms, electrode engineering, endurance/retention, switching dynamics, and neuromorphic functionality, and position their performance within the broader landscape outlined above. Ga_2_O_3_, with its exceptional material properties such as a wide bandgap of 4.9 eV [10], high dielectric strength, and excellent thermal stability, has gained attention as a promising candidate for the next generation of memristor devices [11]. While the research in Ga_2_O_3_-based memristors is still in its infancy compared to more established materials like titanium dioxide (TiO_*x*_), early studies indicate that Ga_2_O_3_ offers unique advantages for devices operating under extreme conditions requiring high endurance. Ga_2_O_3_’s potential for resistive switching behaviour and the ability to incorporate capacitive elements into memristor structures make it an attractive material for advanced applications, including increased data storage capacity and neuromorphic systems. This review provides a comprehensive overview of the current state of research in Ga_2_O_3_-based memristors, focusing on their theoretical foundations, fabrication techniques, and performance characteristics. A particular emphasis is placed on capacitive memristors, which combine the resistive and capacitive properties of materials to enable novel functionalities and enhanced device performance. We discuss the underlying mechanisms of resistive and capacitive switching in Ga_2_O_3_ devices and explore their applications in memory storage, neuromorphic computing, and sensing technologies. Additionally, this work explores challenges and future prospects of Ga_2_O_3_-based memristors, such as the need for precise material control, scalability, and integration with existing semiconductor platforms. Through this review, we aim to provide a foundation for further exploration and development of Ga_2_O_3_-based memristors, paving the way for their integration into emerging electronics.

## 2. General Theory of Memristors

The distinctive characteristic of a memristor is its ability to retain a state of resistance based on the history of the current or voltage applied, even after power is removed [12]. This property makes them highly attractive for applications in non-volatile memory, neuromorphic computing, and programmable electronics [13]. The key properties of memristors that distinguish them from other electronic components include non-volatility and hysteresis. While memristors inherently exhibit non-volatility and hysteresis, similar behaviours are also found in ferroelectric and ferromagnetic systems. However, what distinguishes memristors is that these properties arise directly from electrically driven resistance modulation through ionic migration, defect dynamics, and so on, rather than polarisation reversal or magnetisation switching. In memristors, the hysteresis manifests in the current–voltage domain, enabling simple two-terminal operation and seamless integration into nanoscale electronic circuits. These characteristics make memristors particularly attractive for resistive memory and neuromorphic computing applications. In the case of gallium oxide-based devices, resistance switching is primarily governed by oxygen vacancy migration, which provides a distinct mechanism compared to dipole, or spin-based memory elements [14]. Memristors retain their resistance state after the removal of the driving voltage or current. This property arises from the stable redistribution of ionic or electronic charge carriers within the material and underpins their application in non-volatile memory systems. The current–voltage (I–V) characteristics of memristors exhibit a hysteresis loop, which encodes the device’s memory of past inputs. The shape and area of the loop depend on the material properties, device geometry, and input signal parameters [15]. Memristors can be fabricated at nanoscale dimensions, making them highly suitable for dense memory arrays and compact electronic circuits. Their simple two-terminal structure facilitates integration into existing semiconductor manufacturing processes. Unlike digital memory devices, memristors operate in an analogue manner, allowing them to represent a continuum of resistance states. This feature is critical for applications such as neuromorphic computing, where devices mimic the synaptic weights of biological neurons.

**Figure 2:**
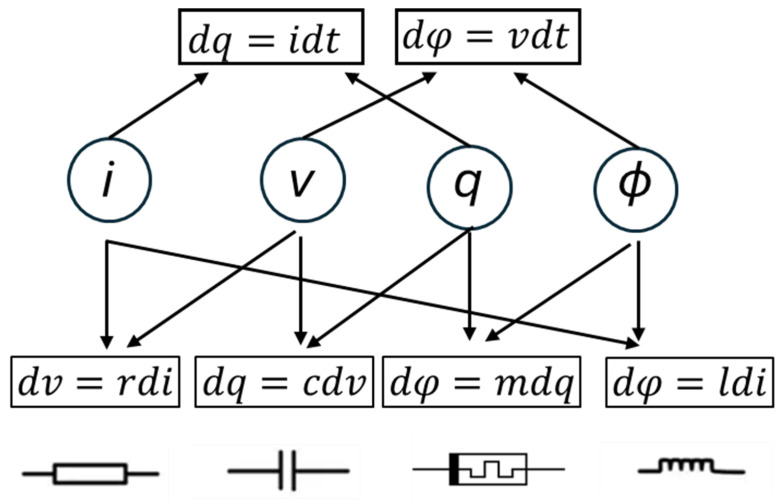
Four fundamental variables in circuit theory.

The theoretical basis of memristors stems from Chua’s hypothesis that a missing relationship existed among the four fundamental variables in circuit theory (shown in Figure 2): voltage (*v*), current (*i*), charge (*q*), and flux linkage ϕ. While resistors relate *v* and *i*, capacitors link *q* and *v*, and inductors connect *i* and ϕ, no direct link had been established between *q* and ϕ. Chua proposed the memristor as the missing element to complete the symmetry. Mathematically, a memristor is defined by

(1) $$M\left(q\right)=\frac{d\phi /dt}{dq/dt}=\frac{v}{i},$$

In Equation (1), *M*(*q*) is the memristance, a charge-dependent resistance. Unlike a resistor’s constant resistance, the resistance of a memristor depends on the charge flow through it, creating a dynamic and nonlinear behaviour. In practical terms, memristors are often described by their current–voltage (i−v) characteristics, which exhibit a hysteresis loop. This loop signifies the device’s memory, as the current through the memristor depends not only on the instantaneous voltage but also on the history of the applied voltage. This property underpins the memristor’s ability to store information and its potential use in memory applications. Since their theoretical inception, various implementations have been realised to achieve monolithic memristor devices. One of the most prominent early monolithic memristor devices was introduced by researchers at HP Labs in 2008, where they presented a solid-state device based on a thin film of titanium dioxide (TiO_2_) [16]. The memristive behaviour arises from the migration of oxygen vacancies, which act as charge carriers, within the TiO_2_ film under an applied electric field. The device comprises two regions: a high-resistance region (depleted of oxygen vacancies) and a low-resistance region (rich in oxygen vacancies). The position of the boundary between these regions determines the overall resistance of the device, which can be modulated by controlling the voltage or current applied. Although early reports of resistive switching random access memory (ReRAM) devices appeared in the late 1990s [17,18] the HP TiO_2_-based device reported in 2008 is often distinguished as the first monolithic memristor implementation. The key distinction lies in the theoretical framework: conventional ReRAM was primarily described as resistive switching memory, whereas the HP work explicitly connected the switching behaviour to Chua’s memristor theory, thereby formalising the device as a fundamental passive circuit element. In other words, while both ReRAM and memristors exploit resistance switching, memristors uniquely embody a direct relationship between charge and flux linkage, offering a unifying theoretical model that conventional ReRAM lacked. HP’s work provided a framework for understanding memristors in terms of ionic drift dynamics. In this model, the rate of change of the boundary position is proportional to the applied electric field, which directly influences the device’s resistance state. The physical dynamics of ion movement, as well as the interplay between drift, diffusion, and redox reactions, form the cornerstone of modern memristor theory. Beyond TiO_2_-based devices, other material systems have been explored for memristive behaviour, including transition metal oxides (HfO_2_, NiO), perovskites, organic polymers, and two-dimensional materials such as graphene and MoS_2_. Each system exhibits unique mechanisms of memristance, ranging from ionic migration and phase changes to electronic effects such as trap-assisted tunnelling and charge storage [19]. The mathematical modelling of memristors extends beyond the static definition of memristance. A generalised memristor can be expressed as given by Chua:

(2) v(t)=M(x)·i(x)

where *x* represents an internal state variable that evolves over time based on the input current or voltage. The evolution of *x* is governed by a state equation (Equation (3)) of the form

(3) $$\frac{dx}{dt}=f\left(x,i\left(t\right),v\left(t\right)\right)$$

where f(x,i(t),v(t)) captures the dynamics of the physical processes governing the memristor’s behaviour. One widely used model incorporates the concept of window functions to constrain the evolution of the state variable within realistic physical bounds. For example, in the HP memristor model, a linear drift assumption is combined with a window function to ensure that the boundary between the high- and low-resistance regions does not move beyond the physical limits of the device. Apart from monolithic memristor development, a cluster of discrete memristor devices have also been developed, for example resistive switching devices based on printed circuit board and ZnO nanowires [20,21,22,23,24]. This category of hybrid devices offers advantages such as easy fabrication and convenient integration into hybrid analogue circuits or sensors.

## 3. Capacitive Memristor Theory

Capacitive memristors are an extension of the traditional memristor concept, which typically involves the relationship between voltage and resistance. The key feature of memristors is their memory property, where the resistance of the device changes based on the history of charge or current passing through it. Capacitive memristors combine this memory-dependent resistance behaviour with capacitive effects, in which the voltage across the device is related to the capacitance change. These devices offer the unique opportunity to explore the interaction between resistance and capacitance, which can lead to novel device behaviours and open up new possibilities for applications in memory, neuromorphic computing, and dynamic signal processing. The fundamental idea behind capacitive memristors is the introduction of a capacitive element into the standard memristor framework. Traditional memristors are purely resistive devices, with the resistance state determined by the charge that has passed through the device. Capacitive memristors, however, involve a coupling between the device’s resistance and capacitance, leading to a more complex relationship between the voltage, current, and charge within the device. This coupling between memory resistance and capacitance allows capacitive memristors to exhibit a broader range of dynamic behaviours that can be used in various types of circuits and systems. To understand capacitive memristors, it is essential to extend the traditional concept of a memristor to include a capacitive term. In a conventional memristor, the voltage across the device is a function of the current and its historical relationship with the charge. The voltage v(t) across a standard memristor is described by Equation (2). In capacitive memristors, the voltage is not only determined by the resistive term but also by a capacitive element, which depends on the charge stored within the device. The voltage v(t) across the device can be described by a more complex equation that includes both the resistive and capacitive terms. The general form of this equation is [25]

(4) $$v\left(t\right)=M\left(q\right)\cdot i\left(t\right)+\frac{1}{C(q)}\cdot q\left(t\right)$$

where M(q) is the memristance, which depends on the charge q(t), and C(q) is the capacitance, which also varies with charge. The term $\frac{1}{C(q)}\cdot q\left(t\right)$ in Equation (4) represents the voltage across the capacitor, which is related to the amount of charge stored on the device. This equation captures the dual behaviour of capacitive memristors, where the voltage is determined by both the resistance and the capacitance of the device, with both properties dependent on the charge state. Capacitive memristors thus combine the fundamental principles of both capacitors and memristors. The resistive part of the device allows for non-volatile memory, where the resistance state depends on the history of charge or current, while the capacitive term introduces dynamic behaviour related to charge storage and the voltage across the capacitor. This combination of resistive and capacitive effects creates a device with more complex and versatile characteristics, making capacitive memristors attractive for a variety of applications. The time evolution of the charge q(t) is related to the current i(t), which is the rate of change of charge in Equation (5):

(5) $$\frac{dq(t)}{dt}=i\left(t\right)$$

The evolution of M(q) and C(q) can be described by state equations that govern how these properties change with the passage of charge. The rate of change of the memristance can be written as

(6) $$\frac{dM(q)}{dt}i\left(t\right)=f\left(M\left(q\right),i\left(t\right)\right)$$

where f(M(q),i(t)) is a function that describes how the memristance evolves based on the current and the history of charge. Similarly, the rate of change of the capacitance can be written as

(7) $$\frac{dC(q)}{dt}i\left(t\right)=g\left(C\left(q\right),i\left(t\right)\right)$$

where g(C(q),i(t)) is a function describing how the capacitance evolves. These state equations (Equations (6) and (7)) capture the memory-dependent behaviour of both the resistance and capacitance of the device. The working mechanism of capacitive memristors is rooted in the interaction between charge carriers and the electric field within the device. Like traditional memristors, capacitive memristors rely on some form of charge storage, such as the migration of defects or ions within a material, However, voltage across the device is influenced by the amount of charge that has been stored. As charge flows through the device, the distribution of charge carriers shifts, which alters the material’s resistance and capacitance. This interaction between charge and the material leads to the resistive switching behaviour observed in memristors, as well as the dynamic capacitance that depends on the amount of charge stored [26]. In capacitors, the capacitance C determines how much charge can be stored for a given voltage, and this capacitance is typically constant for ideal capacitors. However, in capacitive memristors, the capacitance is not constant but instead varies with the charge q(t) that has passed through the device. In practice, capacitive memristors are typically made from materials that can support both resistive and capacitive properties. Many of the same materials used for traditional memristors, such as transition metal oxides [27], can also be used to create capacitive memristors, but with modifications to enhance the capacitive effects. For example, certain oxide materials exhibit both high resistivity and high dielectric constants, which are essential for the capacitive element of the device. By tuning the material properties and device geometry, it is possible to create capacitive memristors with a wide range of performance characteristics, from fast-switching devices to those that can store large amounts of charge.

## 4. Mechanisms in Ga_2_O_3_ Memristors

**Figure 3:**
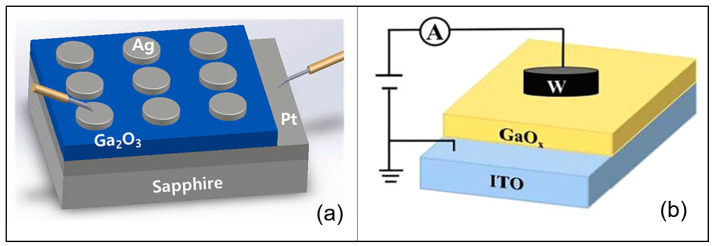
Schematic graphs of Ga_2_O_3_ memristors. (**a**) Ag/Ga_2_O_3_/Pt structure [29].“Reprinted with permission from Ref. [29]. Copyright (2024), Wiley”. (**b**) W/Ga_2_O_3_/ITO structure [30]. “Reprinted with permission from Ref. [30]. Copyright (2024), AIP Publishing”.

Ga_2_O_3_-based memristors typically operate via resistive switching, where the device toggles between a high-resistance state (HRS, “OFF”) and a low-resistance state (LRS, “ON”) under electrical stimuli. The switching can be bipolar (set and reset occur under opposite voltage polarities) or unipolar (set/reset under same polarity with different magnitude). In Ga_2_O_3_ devices, bipolar switching is more commonly observed [28], often attributed to voltage-driven movement of oxygen ions or metallic species. A key factor is the formation and rupture of conductive paths through the insulating Ga_2_O_3_ layer. Typical device architecture for these memristor devices is metal–semiconductor–metal vertical structure (shown in Figure 3). The major device working mechanism of Ga_2_O_3_ based memristors includes conductive filament theory and ionic–electronic conduction theory, with details discussed as below.

### 4.1. Conductive Filament Theory

**Figure 4:**
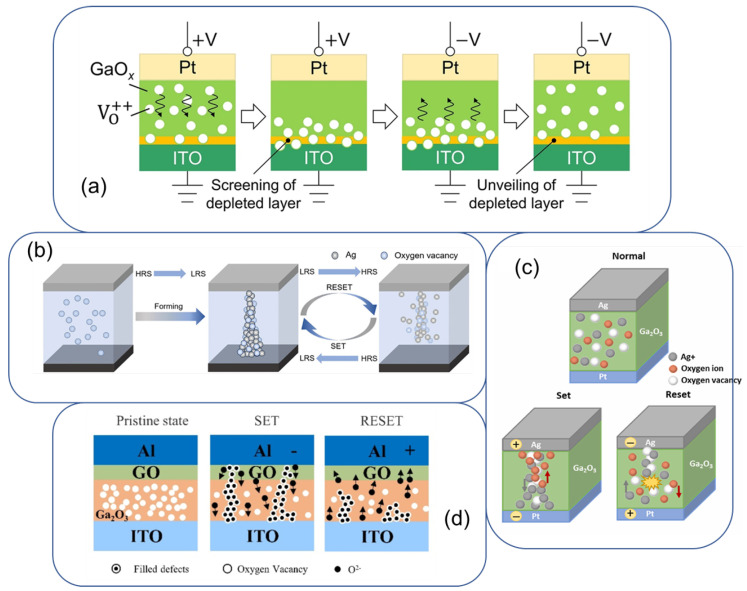
Schematic operating mechanisms of Ga_2_O_3_ memristors. (**a**) Oxygen vacancy re-distribution [31]. “Reprinted with permission from Ref. [31]. Copyright (2023), Springer Nature”. (**b**) Conductive filament forming mechanism [32]. “Reprinted with permission from Ref. [32]. Copyright (2024), Wiley”. (**c**) Conductive filament set and reset process [28]. “Reprinted with permission from Ref. [28]. Copyright (2023), Elsevier”. (**d**) Formation and rupture of the conductive filament model [33]. “Reprinted with permission from Ref. [33]. Copyright (2024), MDPI”.

Most Ga_2_O_3_ memristors show filamentary behaviour, where nanoscale conductive filaments form inside the oxide (several examplary mechanisms are schematically demonstrated in Figure 4). These filaments are believed to consist of aggregated oxygen vacancies (V_*O*_) or metal atoms. When a sufficient electric field is applied (during a “SET” operation), oxygen anions drift away (or cations, leaving behind a chain of V_*O*_ that creates a conductive channel bridging the electrodes [14]. This drastically lowers resistance (LRS). The filament can be dissolved or thinned during the “RESET” (often by reversing bias or increasing it), restoring a higher resistance state as oxygen refills vacancies or metal diffuses out [14]. In Ga_2_O_3_, oxygen vacancies are intrinsic donors and relatively mobile under bias, making filament formation/dissolution a dominant mechanism. For example, Lee et al. observed that increasing oxygen vacancy concentration in Ga_2_O_3_ (by reducing O_2_ during sputter growth) led to lower SET voltages and stronger conductivity in LRS, consistent with filamentary V_*O*_ pathways. Filamentary models also explain the fast-switching speeds attainable—the local conductive path can be formed or ruptured on nanosecond timescales once the field threshold is reached. Filaments may involve metal ions too: in Ag/Ga_2_O_3_/Pt structures, Ag from the top electrode can electrochemically migrate into the oxide under bias, contributing to filament growth. This cation-based switching (often called an ECM mechanism) tends to produce gradual, analogue-type switching because the filament can partially form or dissolve (Ag_+_ movement is more analogue). In fact, the involvement of Ag in filaments was found to smooth the RESET transition (making it gradual rather than abrupt) in annealed Ag/Ga_2_O_3_ devices, which is beneficial for analogue synaptic weight updates. It is worth mentioning that the selection of electrode materials is important for improving the performance of the devices. For example, H. Lee has proved that the Ag+ diffused from the electrode is helpful to form and deform the conduction filament when changing the applied voltage [14].

### 4.2. Ionic–Electronic Conduction Theory

Some studies on Ga_2_O_3_ report non-filamentary or interface-type resistive switching. Here, the change in resistance is more uniformly distributed in the bulk or at the electrode interface, rather than via a narrow filament. Aoki et al. demonstrated that amorphous GaO_*x*_ (x≈1.1) exhibits memristive switching through a bulk mixed ionic–electronic conduction mechanism. Under bias, oxygen ions migrate over a broad region, modulating the entire film’s conductivity in an analogue fashion (without forming a single concentrated filament). This leads to “non-filamentary memristive switching” [34]. In practice, such homogeneous switching appears as a gradual resistance change and often does not require a distinct forming step. The active region might be a Schottky interface: for instance, a Ga_2_O_3_ memristor with a reactive metal electrode could switch by modulating the Schottky barrier via oxygen vacancy drift at the interface. If oxygen vacancies pile up at a Ga_2_O_3_/metal interface, they can lower the barrier (increasing current) and vice versa. Thus, the device behaves like a tuneable diode rather than forming a hard filament. This mechanism can produce analogue resistance updates ideal for neuromorphic synapses, though achieving high ON/OFF ratios may be harder than filamentary devices. Whether a Ga_2_O_3_ memristor switches by filament or interface dynamics often depends on film thickness and defect profiles: ultra-thin (≤5 nm) Ga_2_O_3_ layers or devices with additional layers (such as WO_*x*_/Ga_2_O_3_ stacks [30]) might favour distributed switching since a single filament would essentially short such a thin layer completely. In contrast, thicker or highly defective films lean towards filamentary paths. Notably, both modes rely on oxygen vacancy movement in Ga_2_O_3_, which underscores the material’s ionic aspect. Ga_2_O_3_ memristors primarily leverage oxygen vacancy dynamics—whether coalescing into filaments or modulating interface barriers—to achieve resistive switching. The exact mechanism can be tuned by device structure. Importantly, Ga_2_O_3_’s material properties (wide bandgap, high dielectric strength, and rich defect chemistry) allow these memristive effects to be robust. The wide bandgap ensures low off-state leakage and potentially high ON/OFF ratios [33], while the defect tolerance of Ga_2_O_3_ allows devices to better withstand the repeated cycles involved in switching compared to many other oxides. Almadhoun et al. report >100 cycles with 10^5^ s retention and ON/OFF ratio ≈ 10^8^ [35]; Wang et al. show endurance over 100 cycles in bilayer Al/graphene-oxide/Ga_2_O_3_/ITO RRAM [33]; Lee et al. improve stability via high-temperature annealing in Ag/Ga_2_O_3_/Pt memristors [14]; and Alam et al. achieve ∼300 durable cycles in bilayer β-Ga_2_O_3_/WO_3_/Ag memristors [36]. This characteristic positions Ga_2_O_3_ as a compelling material and shows that with current development, Ga_2_O_3_ memristors already demonstrate more promising results compared to other oxide-based systems such as TiO_2_ (typically 10^2^–10^3^ switching cycles [16]), HfO_2_ (10^4^–10^5^ cycles [27]), NiO (10^2^ switching cycles [2] and ZnO (10^2^–10^3^ cycles [37]), highlighting Ga_2_O_3_’s potential for long-endurance non-volatile memory applications. In both working mechanisms, the intrinsic defect (e.g., oxygen vacancy) is recognised as the key contributor to the resistive switching mechanism. As an emerging material, the density of such defects can be obtained by tuning the stoichiometry between Ga and O, [38] which is an advantage of binary oxide semiconductor materials. It is noticed that other working mechanisms (e.g., space-charged limited carrier transport, phase change, tunnelling etc) are rarely studied in this material, which could be interesting research topics in future.

### 4.3. Electrode Selection

The choice of electrode material strongly influences the resistive switching behaviour of Ga_2_O_3_ memristors. Noble metals such as Pt and Au are typically used as inert electrodes, serving mainly as stable contacts without significantly participating in the switching process. In contrast, active metals such as Ag and Cu can diffuse into the Ga_2_O_3_ layer under bias, forming and dissolving conductive filaments, which leads to electrochemical metallisation (ECM)-type switching with low set voltages but sometimes reduced endurance. Transparent conducting oxides such as ITO are employed to enable optoelectronic or transparent device operation, while transition metals like Ta can serve as oxygen reservoirs, thereby stabilising oxygen vacancy concentrations and improving endurance. Thus, electrode selection not only affects the dominant switching mechanism (filamentary vs. vacancy-driven) but also determines critical parameters such as switching voltage, ON/OFF ratio, endurance, and retention.

## 5. Review of Current Ga_2_O_3_-Based Memristor Devices

Memristors are seen as a potential replacement for flash memory due to their faster switching speeds, lower energy consumption, and greater scalability [39]. Resistive random-access memory (ReRAM) is one of the most studied implementations, with memristors serving as the fundamental switching elements [40]. Memristors are at the forefront of efforts to develop brain-inspired computing systems. Their ability to emulate synaptic behaviour, including plasticity and long-term potentiation or depression, makes them ideal candidates for constructing artificial neural networks. Arrays of memristors can be used to perform in-memory computing, reducing the energy and time costs associated with data movement in conventional von Neumann architectures [19]. While ferroelectric and phase-change memories have been investigated for brain-inspired computing, memristors provide distinct advantages. Phase-change devices rely on thermally driven structural transitions in chalcogenide materials, which often lead to relatively high switching power and slower operation speeds [41]. Ferroelectric devices, although capable of fast switching, typically require more complex device architectures and face scaling limitations due to depolarization fields [42]. In contrast, memristors exhibit simple two-terminal geometry, nanoscale scalability, low power operation, and analogue resistance modulation, making them highly attractive for dense and energy-efficient neuromorphic computing systems [43]. Memristors have been explored for use in analogue circuits, such as filters, oscillators, and adaptive systems [44]. Their tuneable resistance allows for the implementation of reconfigurable and programmable circuits. The unpredictable and nonlinear dynamics of memristors make them attractive for hardware security applications [45], such as physically unclonable functions (PUFs) and random number generators. Ga_2_O_3_-based memristors are still an emerging and relatively uncommon area of research in the broader field of memristor technology. Despite the material’s unique properties and its potential for various high-performance applications, the use of Ga_2_O_3_ in memristor devices is still in its early stages compared to more established materials like titanium dioxide or transition metal oxides [31]. Nonetheless, the exploration of Ga_2_O_3_ for memristor applications holds great promise due to the material’s ability to exhibit resistive switching behaviour, which is fundamental to memristor operation. While the memristive behaviour of Ga_2_O_3_ is less explored than that of other more traditional materials, there is growing interest in its potential for high-performance memory and storage devices. The resistance switching in Ga_2_O_3_ memristors, as with other memristors, can occur in different modes, with the most common being unipolar and bipolar switching [46]. Unipolar switching involves changes in resistance that depend solely on the magnitude of the applied voltage, regardless of its polarity, while bipolar switching requires voltage polarity reversal to toggle between resistance states. While unipolar switching has been widely observed in memristive devices, bipolar switching tends to be more common in many memristors due to its controlled nature and reversible switching behaviour. Ga_2_O_3_-based memristors have not been thoroughly researched with promising results being limited in the field. Table 1 gives sight to key properties exhibited by the few Ga_2_O_3_-based memristors currently available.

In reviewing this field, several trends and insights emerge. First, film phase and quality strongly influence performance. Amorphous Ga_2_O_3_ is often intentionally used for its high initial resistance, adjustable Ga/O stoichiometry and large bandgap, which tend to yield higher ON/OFF ratios [33]. For example, Wang et al. found (amorphous gallium oxide) a-Ga_2_O_3_ to be more suitable than β-Ga_2_O_3_ for RRAM because of its lower leakage current and sensitivity to oxygen content. Their bilayer Al/GO/Ga_2_O_3_ device achieved ON/OFF > 10^3^ and stable 10^4^ s retention without needing any post-fabrication forming step. On the other hand, improving crystallinity via annealing can enhance stability: Lee et al. showed that recrystallising sputtered Ga_2_O_3_ into β-phase (by 600–800 °C anneals) reduced the leakage in HRS and thereby increased the HRS/LRS ratio [14]. Their annealed devices reached ON/OFF in the 10^3^ range, higher than the ∼10^2^ of the as-deposited amorphous devices. Thus, while a fully amorphous film gives high resistance contrast, moderate crystallinity can help consistency and lower set/reset voltages. It appears that an optimal Ga_2_O_3_ microstructure for memristors lies somewhere between highly defective and highly ordered—enough defects to enable switching, but not so many as to cause excessive variability or low endurance.

Another trend is the critical role of oxygen vacancies. Virtually all studies pinpoint oxygen vacancy migration as the key switching mechanism in Ga_2_O_3_. Controlling the oxygen stoichiometry during growth or via doping can tune the switching. In sputtered Ga_2_O_3_, adding oxygen gas during deposition reduces the vacancy density and increases the SET voltage (or even suppresses switching if too stoichiometric) [28]. Conversely, oxygen-poor growth yields filament-rich devices that switch at lower voltages but may suffer from variability. Post-growth annealing in certain atmospheres can also modulate vacancies—annealing in vacuum or forming gas would add vacancies, whereas oxidising anneals could quench some vacancies. The literature indicates that devices with moderate oxygen vacancy concentrations perform best. Excess vacancies cause many random filaments leading to cycle-to-cycle variability, while too few vacancies make forming a filament difficult (high forming voltage).

However, a lateral co-planar ITO/Ga_2_O_3_/ITO structure was demonstrated in [55]. The lateral device showed memristive hysteresis up to ∼±20 V sweeping, where switching occurred at relatively high voltages (∼7 V). Vertical MIM devices usually achieve better ON/OFF at lower voltages (a few volts) because the electric field is uniform across a thin film. The lateral device structure exhibited a capacitance-coupled effect, where the large geometric capacitance formed between the interdigitated electrodes introduced an additional capacitive current component. This capacitive contribution effectively modified the charging and discharging dynamics during voltage sweeps, which in turn altered the slope and width of the observed I–V hysteresis loop. This underscores that in thin-film Ga_2_O_3_ memristors, both resistive and capacitive effects can co-occur, effectively making the device a memcapacitor as well as a memristor.

Comparing Ga_2_O_3_ memristor performance to other oxide memristors, we see competitive metrics in some aspects and gaps in others. ON/OFF ratios in the 10^2^–10^3^ range are routinely achieved [33], which is on par with HfO_2_ or TiO_2_-based RRAM. The retention times (often 10^4^ s at room temperature) indicate stable non-volatile behaviour [33]. For instance, using graphene oxide (GO) in the bilayer GO/Ga_2_O_3_ device held multiple resistance levels for 10,000 s without decay. However, endurance (number of switching cycles) is an area for improvement. Reports show endurance of only 10^2^–10^3^ cycles before failure [56]. This is lower than mature RRAM materials (which can reach 10^6^ cycles). The endurance limitations in Ga_2_O_3_ memristors often relate to filament overgrowth or permanent damage in the oxide after repeated high-field stressing. Approaches like the GO buffer layer, which limited and stabilised filament formation, have improved endurance five-fold (from ∼20 to >100 cycles) by acting as an oxygen reservoir to heal the oxide [33]. Nonetheless, further work is needed to reach industrial-level endurance.

**Figure 5:**
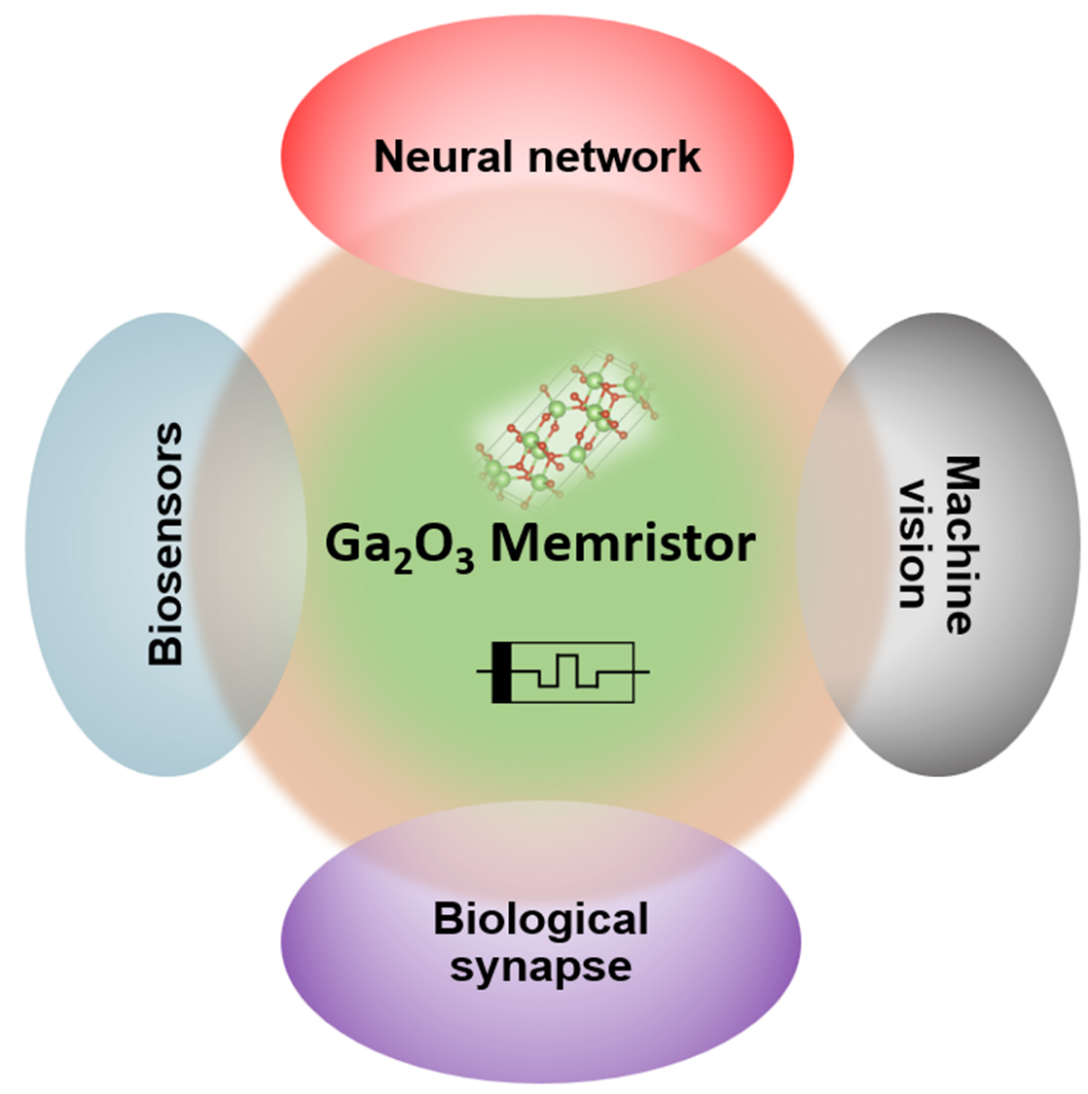
A schematic of Ga_2_O_3_-based memristor used in various applications.

Another notable observation is Ga_2_O_3_ memristors’ capability for multi-level and analogue switching. Unlike binary digital memory, analogue switching is desired for synaptic weight storage in neuromorphic computing. Several Ga_2_O_3_ devices intrinsically exhibit gradual set and reset behaviour, especially those involving cation migration (Ag, or the Al/GO case where an AlO_*x*_ interfacial layer forms). Recent development on memristive devices being used in neuromorphic system has been described in [57], where each memristor acts as a neuron. Figure 5 shows that Ga_2_O_3_-based memristors have several applications including biosening, biological synapse, machine vision, and neural network.

In summary, literature results confirm that Ga_2_O_3_-based memristors can achieve high resistance ratios, long retention, and functional analogue switching. The combination of a wide bandgap and mobile oxygen vacancies in Ga_2_O_3_ is key to these properties. Performance is still variable across reports, influenced by factors like film deposition method (affecting phase and defects), device structure, and electrode materials. Switching speed is another key performance parameter for memristive devices. However, in the case of Ga_2_O_3_-based memristors, only limited studies have so far reported quantitative switching speed data. As a result, while we acknowledge its importance for benchmarking, comprehensive comparisons are not yet possible due to the scarcity of systematic studies in the literature. Future investigations will be essential to establish a clearer picture of switching dynamics in Ga_2_O_3_ memristors.

## 6. Future Prospects

The continued advancement of memristor technology is driven by progress in materials science, device engineering, and computational modelling. As researchers push the boundaries of what these devices can achieve, several emerging directions are gaining momentum. One major focus is the development of new materials [58] and composite structures that offer improved performance, enhanced stability, and greater energy efficiency. Two-dimensional materials and semiconductor heterostructures are particularly promising due to their tuneable properties and compatibility with nanoscale fabrication [59]. Another important area involves designing memristive devices that combine resistive switching with additional functionalities, such as ferroelectric behaviour [60], magnetic coupling, or optical response [29]. These multifunctional devices hold potential for next-generation applications, including adaptive electronics, smart sensors, and reconfigurable logic. In parallel, efforts are being made to integrate memristors with conventional CMOS technologies, enabling the creation of hybrid systems that can harness the strengths of both traditional and emerging components. Such integration is especially relevant for improving the energy efficiency of artificial intelligence accelerators and neuromorphic computing platforms. Within this broader landscape, Ga_2_O_3_ is emerging as a highly promising material for memristor development. Its ultra-wide bandgap, high dielectric strength, and exceptional thermal stability position it as an ideal candidate for devices operating under extreme conditions [31,61]. Ga_2_O_3_-based memristors can tolerate high voltages and temperatures, making them suitable for applications where reliability and endurance are critical. Additionally, the ability to fabricate high-quality Ga_2_O_3_ thin films with uniformity supports scalable device manufacturing, which is essential for commercial deployment. Beyond its physical robustness, Ga_2_O_3_ enables fast switching speeds and high endurance, both of which are vital for non-volatile memory, neuromorphic systems, and advanced sensing technologies [53]. As fabrication techniques mature and the fundamental mechanisms of Ga_2_O_3_-based memristors become better understood, the prospects for low-power, high-density, and durable electronic systems are increasingly within reach. Whether in edge computing, smart sensing, or AI acceleration, Ga_2_O_3_ holds significant potential to shape the future of electronics and contribute meaningfully to the evolution of next-generation devices.

## 7. Challenges

One of the challenges that researchers face with Ga_2_O_3_-based memristors is that the material itself is not as commonly used in memristor applications, which limits the number of studies and practical devices based on it. This relative scarcity of research means there is still much to understand about the optimal conditions for producing reliable, repeatable switching behaviour in Ga_2_O_3_ memristors [53]. The fabrication techniques, for example, are still being refined to produce high-quality, uniform films of Ga_2_O_3_ that exhibit consistent switching characteristics. Moreover, the interactions between Ga_2_O_3_ and electrode materials are still being studied to understand how to best control the movement of defects and ensure long-term stability and endurance of the devices. Despite these challenges, Ga_2_O_3_ memristors are an exciting area of research, especially in the context of high-temperature and high-voltage applications where traditional materials may not be suitable. Their potential for non-volatile memory and neuromorphic computing applications, where devices need to mimic the behaviour of biological neurons, makes them a topic of significant interest in the scientific community. As research progresses, it is likely that Ga_2_O_3_ will emerge as a viable candidate for memristive devices, but much remains to be explored in terms of materials science, fabrication techniques, and device optimisation. In the future, as more researchers delve into this area and overcome existing challenges, Ga_2_O_3_ could provide an alternative to current memristor technologies, with its own unique set of advantages and applications. Despite their promising potential, capacitive memristors face several challenges in terms of practical implementation. One of the primary difficulties is the need for precise control over both the resistive and capacitive properties of the device [62]. The materials used for capacitive memristors must be carefully selected to ensure that both resistance and capacitance can be tuned independently and reliably. Moreover, the modelling and simulation of capacitive memristors are more complex compared to traditional memristors, as the device behaviour depends on both resistive and capacitive terms. Developing accurate models for capacitive memristors, and understanding how they interact with various input signals, is essential for optimising their performance in real-world applications. The unique properties of capacitive memristors make them a promising candidate for a wide range of applications. As materials and fabrication techniques continue to improve, capacitive memristors could become an integral part of future memory and computing systems, offering enhanced performance and versatility. Despite the promising properties of Ga_2_O_3_ memristors, their fabrication remains a significant challenge. Most current research emphasises crystalline phases of Ga_2_O_3_ (particularly β-Ga_2_O_3_), where established growth techniques such as molecular beam epitaxy (MBE) and pulsed laser deposition (PLD) provide high-quality films. In contrast, there are relatively few systematic studies on the processing of amorphous Ga_2_O_3_, even though amorphous films could offer advantages such as low-temperature deposition, large-area scalability, and potentially more uniform switching behaviour. Furthermore, the range of fabrication methods explored for Ga_2_O_3_ memristors is still narrow compared to other oxide systems: most reports rely on sputtering or PLD, with limited exploration of solution-based routes, atomic layer deposition, or chemical vapor deposition. This lack of diversity in processing strategies restricts our understanding of how deposition conditions, microstructure, and defect chemistry influence resistive switching, and underscores the need for more comprehensive studies on amorphous Ga_2_O_3_ and alternative fabrication techniques. There is also little to no information in the field for processing amorphous material using wet or dry etching, which is vital in order to fabricate repeatable devices in methods such as lithography.

## 8. Conclusions

Ga_2_O_3_-based memristors represent a promising frontier in the evolution of next-generation electronic devices, offering a unique combination of high thermal stability, wide bandgap properties, and manageable defect chemistry. This review has outlined the theoretical foundations of memristors, explored the mechanisms of resistive and capacitive switching, and highlighted the distinctive advantages Ga_2_O_3_ brings to the memristor landscape. Although Ga_2_O_3_ is still an emerging material within the memristive domain, early experimental results demonstrate its potential for high-performance applications, particularly in harsh environments and neuromorphic systems. Key challenges remain, especially in the areas of material synthesis, device endurance, and integration with existing semiconductor platforms. The dual-mode switching capabilities and analogue behaviour exhibited by Ga_2_O_3_ memristors present exciting opportunities for multifunctional memory and in-memory computing architectures. As fabrication techniques improve and our understanding of defect dynamics deepens, Ga_2_O_3_ is likely to play a critical role in enabling more energy-efficient, scalable, and adaptive electronic systems. Continued interdisciplinary research across materials science, device engineering, and modelling will be essential for unlocking the full potential of Ga_2_O_3_ memristors in both emerging and conventional applications.

## Figures and Tables

**Table 1:** Comparison of various gallium oxide memristors.

Electrode Material	Ga_2_O_3_ Thickness (nm)	Deposition Method	On/Off Ratio	Endurance (Cycles)	Retention (s)	Ref
Pt/Ga_2_O_3_/ITO	-	PLD	10	100	-	[31]
Pt/Ga_2_O_3_/SiC/Pt	-	PLD	10^3^	80	10^4^	[47]
Au/Ga_2_O_3_/Au	-	VLS	10	200	10^3^	[48]
Pt/Ga_2_O_3_/ITO	90	PLD	10^2^	30	-	[34]
Pt/Ga_2_O_3_/Pt	100	RF sputtering	-	100	-	[49]
Pt/Ga_2_O_3_/Pt	100	RF sputtering	-	100	-	[50]
Pt/Ga_2_O_3_/Pt	100	RF sputtering	10^3^	100	10^4^	[29]
Ag/Ga_2_O_3_/Pt	100	RF sputtering	10^8^	30	-	[14]
W/Ga_2_O_3_/ITO	7	RF sputtering	-	-	-	[51]
ITO/Ga_2_O_3_/Pt/Ti	70	PLD	10	-	10^4^	[52]
Au/Ga_2_O_3_/WO_3_/Ag	80	E-beam evaporation	182	300	10^3^	[36]
Ta/Ga_2_O_3_/Pt	20	RF sputtering	50	3 × 10^6^	10^4^	[53]
Au/Ti/Ga_2_O_3_/Ti/Au	-	MOCVD	-	-	-	[54]
Al/GO/Ga_2_O_3_/ITO	15	RF sputtering	10^3^	100	10^4^	[33]

## Data Availability

Dataset available on request from the authors.
